# A Treatise on Diphtheria

**Published:** 1887-12

**Authors:** 


A TREATISE ON DIPHTHERIA, INCLUDING CROUP, TRACHEOTOMY AND INTUBATION. By Dr. A. Sanne. Translated by Henry Z. Gill, A. M., M. D., LL. D., St. Louis, Mo: J. H. Chambers & Co. 1887.

This book of six or seven hundred pages has been for several weeks upon our table awaiting such notice or review as we might be able to give it. Other engagements have prevented an earlier reference to it, and must still limit us to little more than an earnest commendation of it to our readers and to the entire medical profession.

The scope and design of the work are expressed in its title; a perusal of it shows that its objects have been carefully, laboriously and successfully accomplished, and we have in it by far the most perfect monograph hitherto given to the public upon the important subject of which it treats.

Between fifty and one hundred thousand of the people of the United States die annually of diphtheria, and from the most accurate statistics obtainable, at least one thousand in the State of Georgia. The confessed contagiousness of the disease, besides its fatality, adds to the interest and apprehension with which it is regarded by the profession and by the community.

The history of this disease, from a remote period under different names, as cynanche, anginosa, croup, diphhirite and diphtheria, is graphically and instructively given, as well as the shifting phases of opinion of its etiology, pathology and treatment.

While upon many points of detail there exist great uncertainty and dispute, there appears to be a general concurrence of opinion that diphtheria is a general infectious and contagious disease, of which the local manifestations, presenting themselves upon the mucous membrane or skin of different parts of the body, are only troublesome complications demonstrating its septic nature.

The treatment of the disease naturally is divided into general and local medication, to each of which division attention is directed, the relative importance and value of each impartially discussed and apparently just conclusions drawn.

If our space permitted it would be useful to present liberal quotations from this part of the work, but they would poorly supply the place of the entire book which demands an honored position in every physician's library.

